# Supplementary material for: Pre-plaque conformational changes in Alzheimer's disease-linked Aβ and APP
Source: Nat Commun. 2017 Mar 13;8:14726. doi: 10.1038/ncomms14726 (PMC5355803; doi:10.1038/ncomms14726)
Supplement: Supplementary Information — Supplementary Figures, Supplementary Table and Supplementary References [file ncomms14726-s1.pdf]

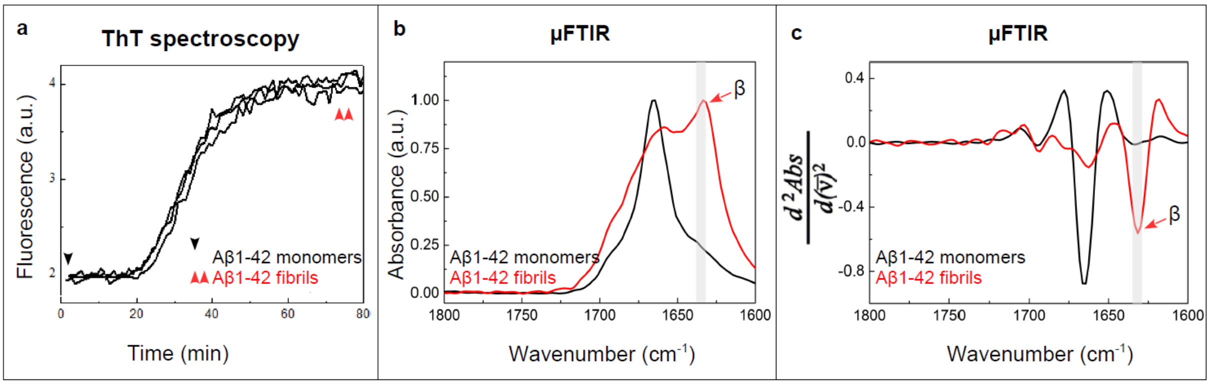

Supplementary Figure 1. Sample preparation used for μFTIR does not induce artificial β-aggregation. (a) Thioflavin T (ThT) kinetic assays of 20 μM Aβ1-42 fibril formation in the presence 6 μM ThT at 37°C. Since ThT fluorescence intensity at 480 nm is proportional to the mass of amyloid fibrils, the increase of ThT fluorescence shows that after several hours of incubation, Aβ1-42 formed β-sheet fibrils. The ThT fluorescence experiments were repeated three times. In parallel to the ThT kinetic assays, Aβ1-42 was incubated without adding ThT in low-bind Eppendorf tubes at 37°C under quiescent conditions. For μFTIR, 5 μL drops of samples of monomeric and fibrillar fractions were placed on CaF_2 spectrophotometric windows, and then all samples were snap frozen and cryo-dried. Monomeric Aβ1-42 was collected at 4°C, before the temperature switch. Fibrillar Aβ1-42 were collected after ThT fluorescence reached a plateau indicating the presence of mature fibrils in the suspension as indicated by red arrows. **(b)** Normalised representative μFTIR absorbance spectra taken from monomeric and fibrillar Aβ1-42 samples. Background spectra were collected from a clean area of the same CaF_2 window. **(c)** As shown by second derivatives of FTIR spectra, a characteristic peak for β-sheet structures was evident only for fibrillar Aβ1-42 (grey bar) but not for monomers of Aβ1-42, supporting that the sample preparation for μFTIR does not introduce artificial β-sheet formation. Experiments were repeated for 3 independent sample preparations.

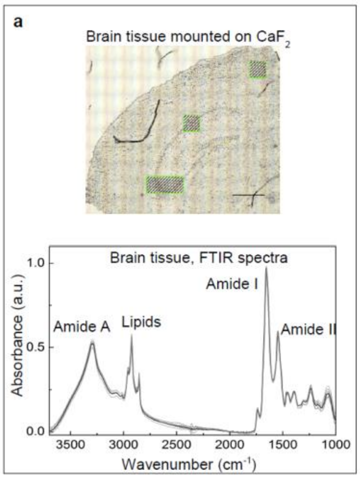

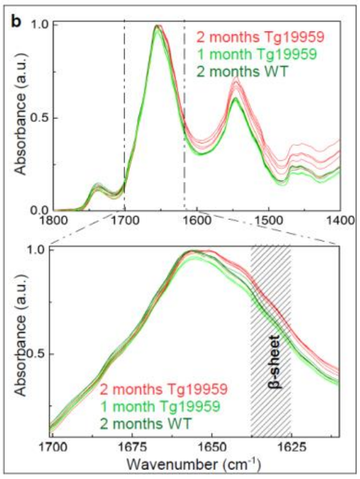

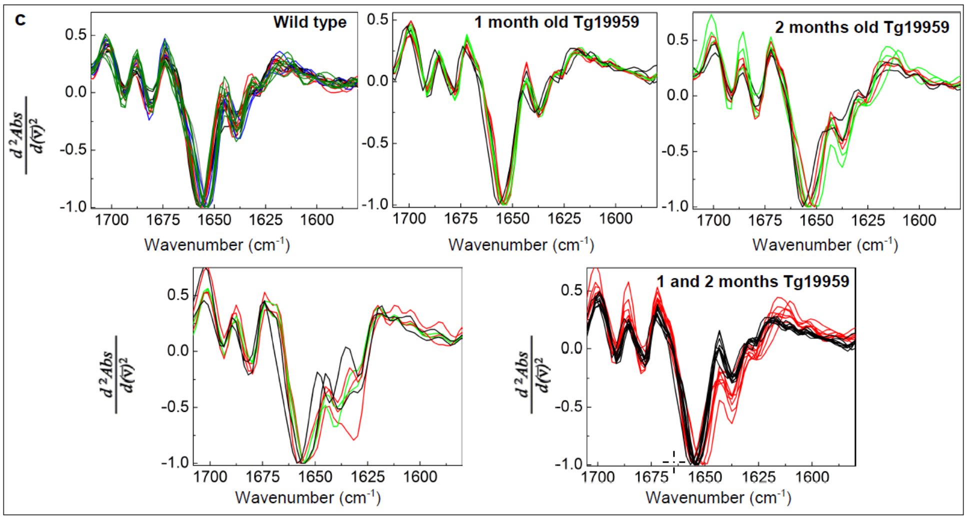

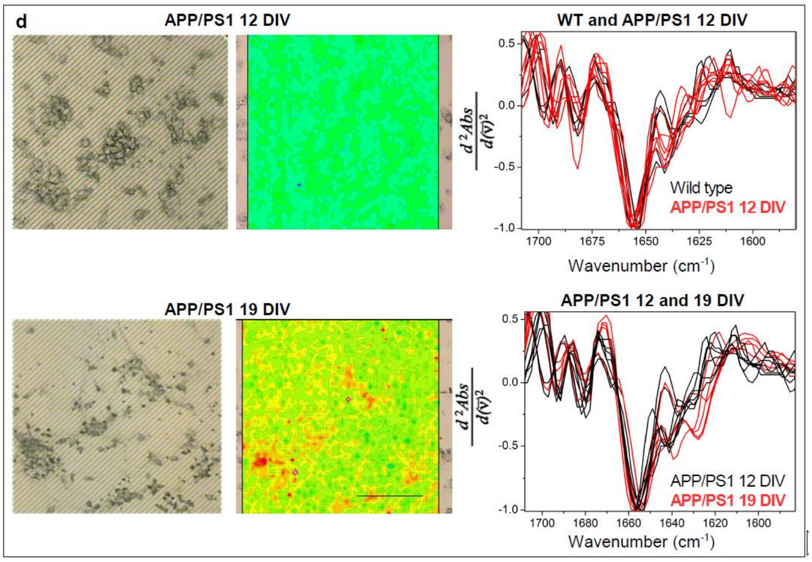

Supplementary Figure 2. FTIR absorbance spectra of Tg19959 and wild-type mouse brain sections. (a) An overview of a brain tissue slice placed on a CaF_2 window (upper panel). Brain tissue was snap frozen, cryostat sectioned, mounted on CaF_2 windows, and stored at -80°C until use. Dashed squares show the positions of μFTIR measurements. Scale

bar is 700 µm. Lower panel: Baseline corrected and normalized FTIR absorbance spectra of mouse brain tissue recorded with an aperture of 8×8 µm^2; the instrument resolution was set at 4 cm^{-1}; 250 - 1000 co-added scans per spectrum (5 to 10 spectra per age) **(b)** Baseline corrected and normalized FTIR absorbance spectra of Tg19959 and wild-type mice. A shoulder corresponding to β-sheet structures is indicated by a grey bar. **(c)** Normalised 2nd derivatives of FTIR spectra from brain tissue, spectra of one colour corresponds to one animal. **(d)** Left panels: an overview of a neuron grown on a CaF_2 window (left panels). Neurons were fixed and stored at -80°C until use. Middle panels: µFTIR maps integrated for the β-sheet spectral region at 1640-1620 cm^{-1} show absorption intensities for the β-sheet content in AD transgenic neurones at different times in culture, β-sheet content appears as red spots. Scale bar is 100 µm. Right panels: Normalised 2nd derivatives of FTIR spectra taken from APP/PS1 neurons at 12 and 19 DIV, and wild-type neurons at 19 DIV (panels 3 and 5).

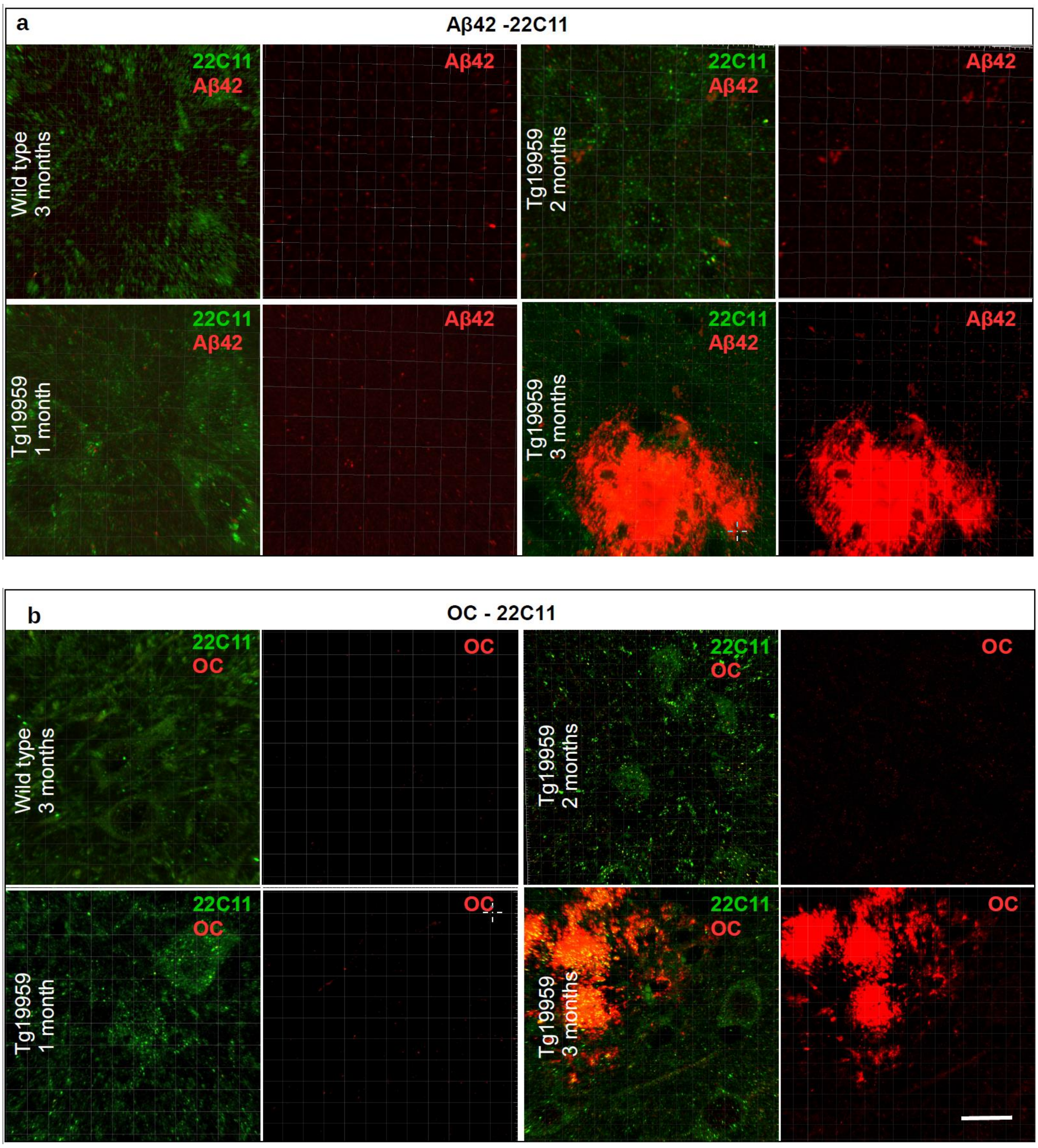

Supplementary Figure 3. Aβ42 and amyloid β-aggregation in brain tissue of Tg19959 mice. (a) Aβ42 in brain tissue with age as revealed by an Aβ42 specific antibody (Invitrogen 700254). **(b)** Amyloid fibrils in Tg19959 brain tissue revealed by OC antibody. OC positive amyloid plaques are visible in Tg19959 mouse brain tissue at the age of 3 months. Wild-type mouse brain was set as a threshold. Neuronal cells are visualised using antibody 22C11 against the N-terminus of APP. Images are representative, N=3 per genotype/age. Scale bar is 10 µm.

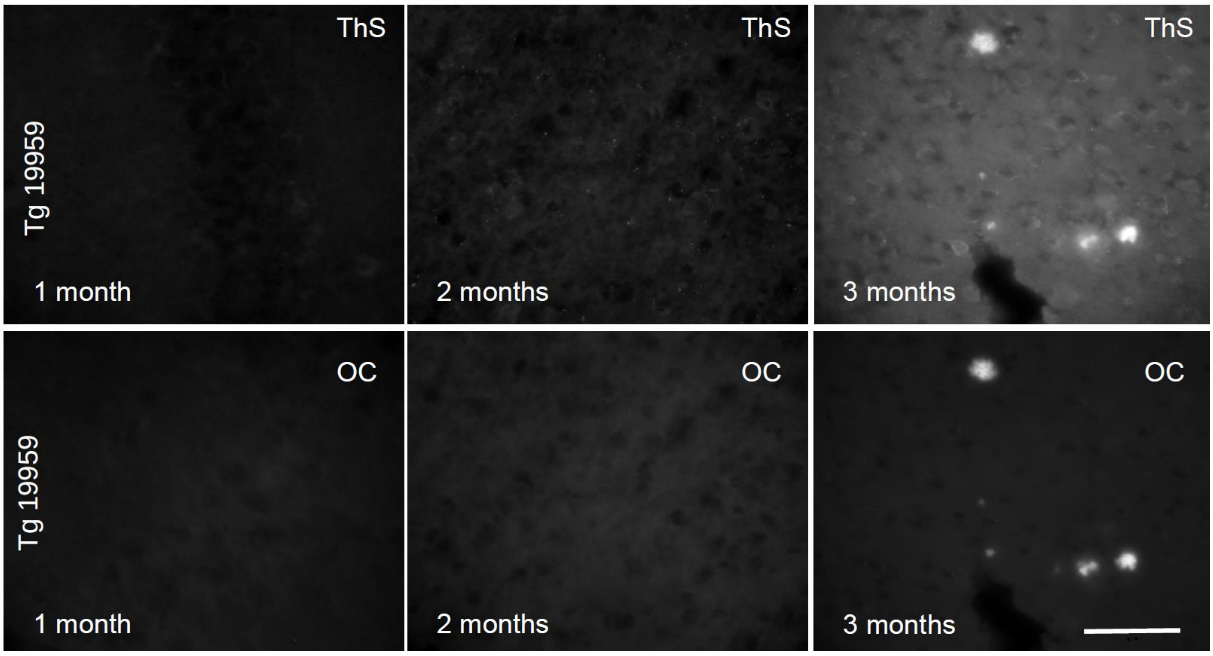

Supplementary Figure 4. Amyloid-β fibrillization in brain tissue of Tg19959 mice. Adjacent sections of the brain tissue sections used for FTIR measurements were double-labelled with OC and ThS. Double labelling visualised amyloid plaques in Tg19959 mouse brain tissue only at the age of 3 months. Scale bar is 50 µm. Wild-type mouse brain was set as a threshold. Images are representative, N=3 per genotype/age.

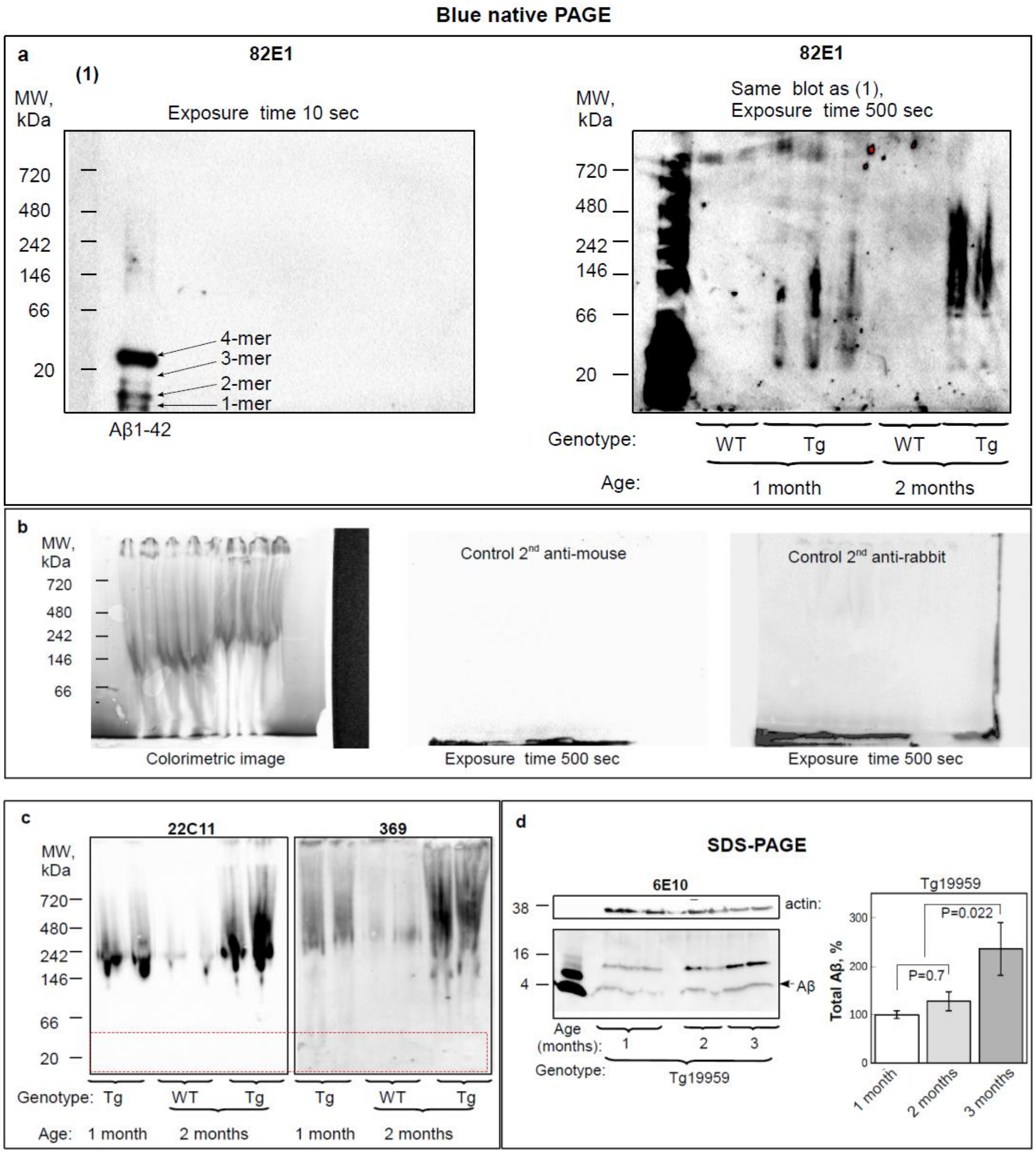

Supplementary Figure 5. Un-cropped versions of BN-PAGE western blots shown in Fig 4. (a) BN-PAGE and subsequent Western blot of membrane-associated TBST fractions of wild-type and Tg19959 mouse brain homogenates at 1 and 2 months of age. Aβ detected by human specific 82E1 antibody as shown in underexposed (1) and overexposed (2) versions. Synthetic human Aβ1–42 peptide was used as a size marker and positive control. **(b)** No cross-reactivity is evident as revealed by a WB with only secondary antibodies. **(c)** Un-cropped blots showing that the LMW Aβ band does not correspond to any APP fragments as revealed by antibodies against the APP N- (22C11) and C- (369) termini (red box). **(d)** As expected, SDS-PAGE shows increasing amounts of Aβ (arrowhead) in protein extracts from Tg19959 mouse brains as revealed by antibody 6E10. Statistical analysis of monomeric Aβ in brain homogenates is with ANOVA ($P < 0.01$) followed by *Bonferroni's post-hoc* comparisons test ($P < 0.01$); mean ± SD. (N = 4 per genotype/age).

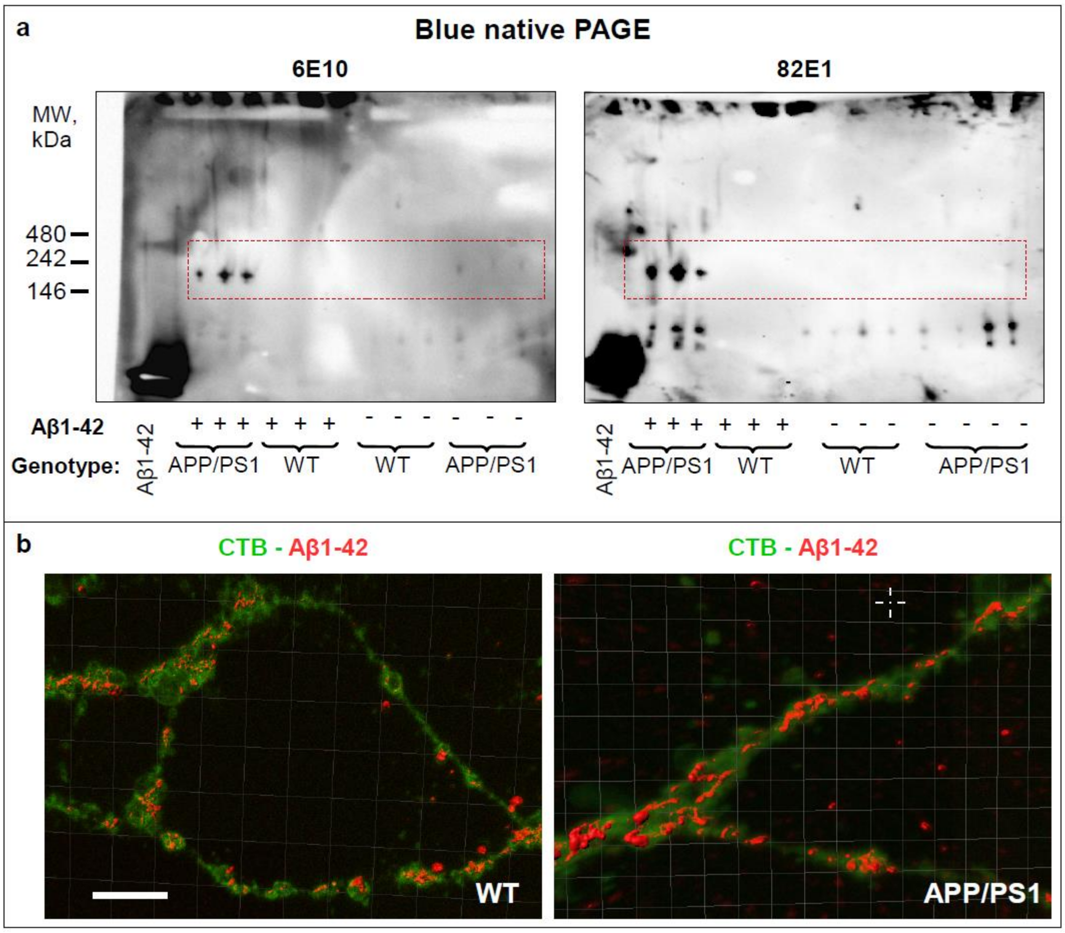

Supplementary Figure 6. Role of APP in Aβ binding. (a) Wild-type and APP/PS1 primary neurons were treated with 1 μM of Aβ1-42 for 24 hours. The added Aβ1-42 was detected by BN-PAGE followed by Western blot with human-specific Aβ antibody 82E1 and human-specific Aβ/APP antibody 6E10; bands which are consistent with the molecular weight of APP are shown in the red boxes, N=3 embryos. **(b)** Immunofluorescent confocal microscopy images of wild-type and APP/PS1 primary neurons treated with 1 μM Aβ1-42 for 1 hour. Untreated neurons were set as a threshold. Aβ1-42 was detected by antibody 82E1 (red). Neuronal membranes were stained with the dye Cholera toxin subunit B1 (CTB) specific to GM1 gangliosides abundantly present in the cell membrane (green); scale bar is 1 μM.

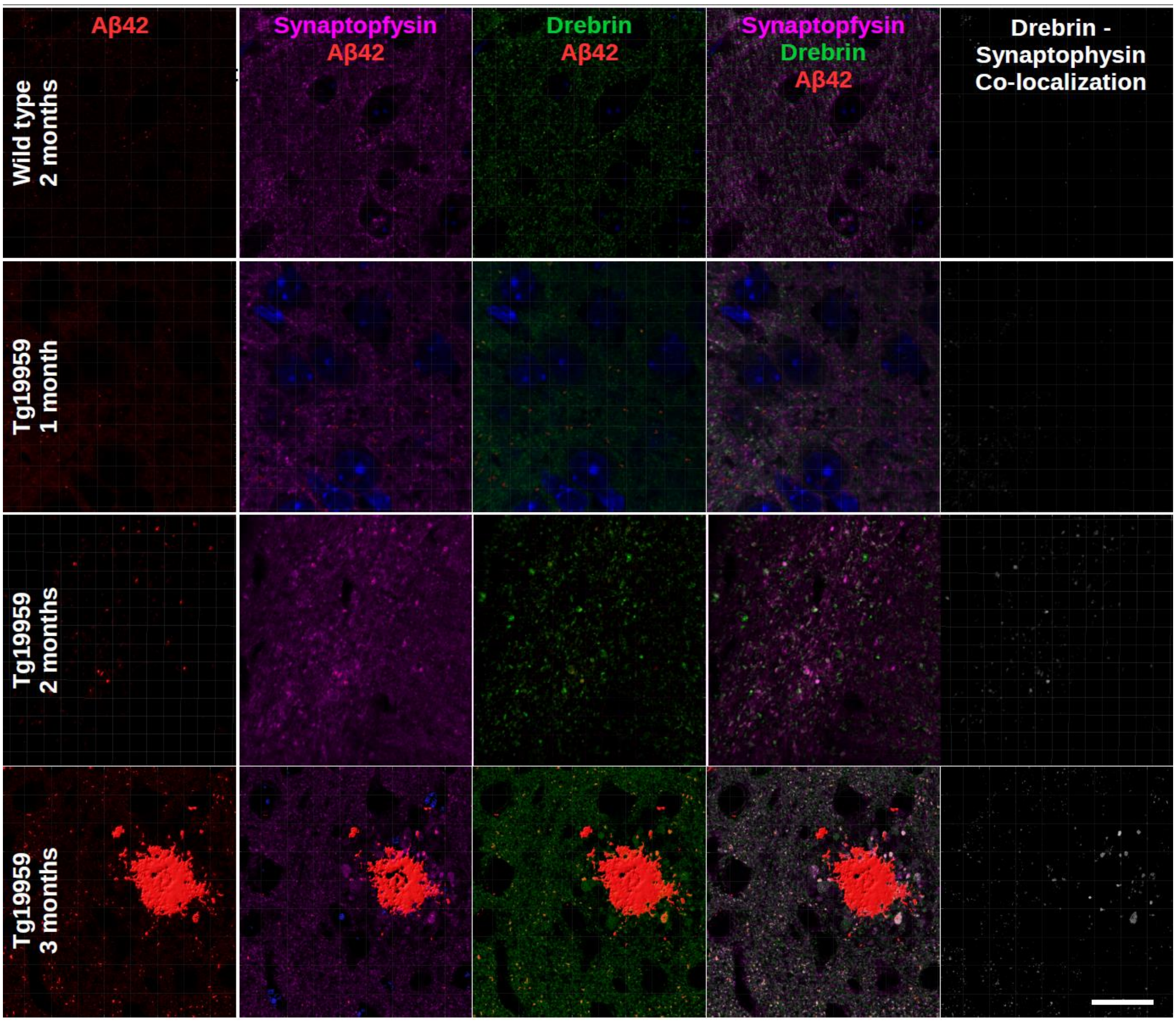

Supplementary Figure 7. Aβ42 alters the cytoarchitecture of synaptic terminals in Tg19959 mouse brains with age. Aβ42 in brain tissue slides was labelled with an Aβ42 specific antibody (Invitrogen 700254) and is shown in red; at 3 months an amyloid plaque is clearly evident, while punctate Aβ42 increases are detectable already at 2 months. The middle panels show the pre- synaptic marker synaptophysin (magenta), the post-synaptic marker drebrin (green) and Aβ42 (red). The white puncta in the far right panel shows co-localization of drebrin and synaptophysin, which is evident in 2 and 3 month-old Tg19959 mice. Around the plaque in the 3-month-old Tg19959 mouse, the locations of these even more enlarged puncta (compared to at 2 months) are consistent with that of dystrophic neurites. Images are representative, N=3 per age/genotype. Scale bar is 10 µm.

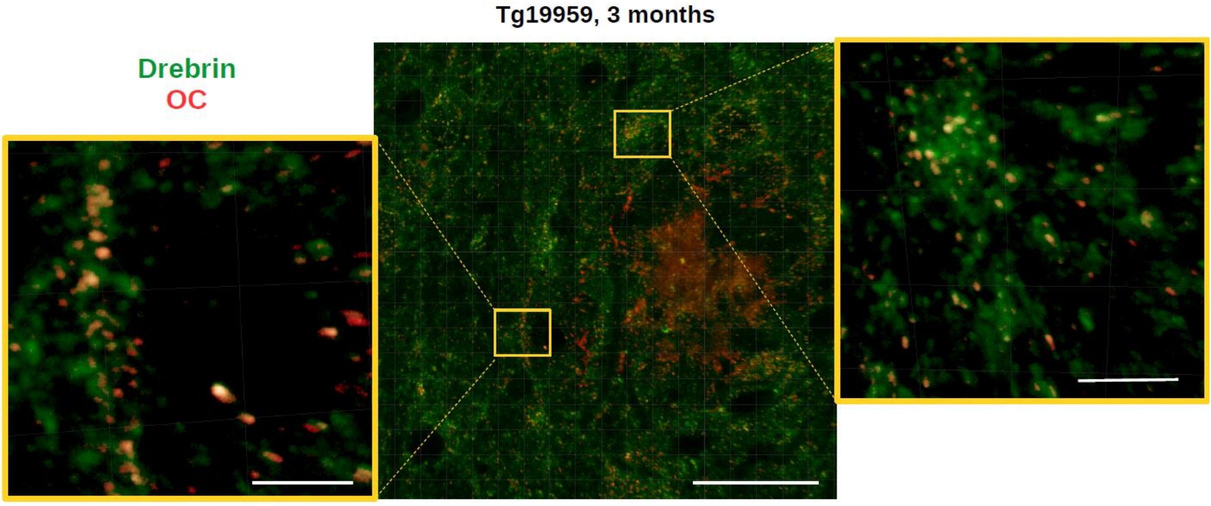

Supplementary Figure 8. Amyloid aggregation in dendritic terminals of Tg19959 mouse brains. Double labelling with OC and the post–synaptic marker drebrin (green) revealed amyloid fibrils (OC, red) co-localized (yellow) with the post-synaptic protein drebrin (green) at the age of 3 months. Wild-type mouse brain was used to set the threshold. Images are representative, N=3. Scale bars: left panel: 1.5 µm, middle panel: 10 µm, right panel: 2µm.

Supplementary Table 1. List of primary antibodies

Antibody	Target Epitope	Species and type	Dilution (WB)	Dilution (IF)	Source	Cat. #
369	Human/mouse full length APP, α/βAPP, α/β CTF, APP C-terminus	Rabbit polyclonal	1:1000		Buxbaum et al. (1990)[1]	
6E10	Human Aβ, full length APP, α/βAPP, α/β CTF, a.a. 3-8 of Aβ	Mouse monoclonal	1:1000		BioLegend	Previously Covance catalog# SIG-39320
22C11	Human APP, N-terminus, a.a. 66-81 of APP	Mouse monoclonal	1:1000		Merck Millipore	ab348
P2-1	Human APP, N-terminus, a.a.104-118 of APP	Mouse monoclonal	1:1000		ThermoFisher	OMA1-03132
82E1	Human Aβ1-x, N-terminus specific, a.a. 1-4 of Aβ	Mouse monoclonal	1:1000	1:200	IBL International	10323
12F4	Human/mouse Aβx-42, C-terminus specific	Mouse monoclonal	1:1000	1:200	BioLegend	Previously Covance catalog# SIG-39142
MBC Aβ42	Human/mouse Aβx-42, C-terminus specific	Mouse monoclonal	1:1000		Kindly provided by Dr. Haruyasu Yamaguchi[2]	
Aβ42	Human/mouse Aβx-42, C-terminus specific	Rabbit polyclonal		1:200	Invitrogen	700254
OC	Amyloid fibrils	Rabbit polyclonal		1:1000	Merck Millipore	ab2286
Synaptophysin	Synaptophysin			1:1000	BioLegend	MAB5258
Drebrin	Drebrin	Rabbit polyclonal		1:1000	Abcam	ab11068
Cholera toxin Subunit B	Ganglioside GM1	Alexa Fluor 488 conjugate		1:1000	Molecular probes	C-34775
Map2	Map2	Chicken polyclonal		1:1500	Abcam	ab5392

Supplementary References

1. Buxbaum, J. D. *et al.* Processing of Alzheimer beta/A4 amyloid precursor protein: modulation by agents that regulate protein phosphorylation. *Proc Natl Acad Sci USA* 87, 6003–6006 (1990).

2. Takahashi, R. H. *et al.* Intraneuronal Alzheimer Aβ42 accumulates in multivesicular bodies and is associated with synaptic pathology. *Am. J. Pathol.* 161, 1869–1879 (2002).
